# Clinical Application of the Bilateral Abalakov Technique in Presurgical Orthodontic Decompensation: A Case Report

**DOI:** 10.7759/cureus.89680

**Published:** 2025-08-09

**Authors:** Pierre Sockalingum, Louis Lespinasse, Adrien Leporcq, Ludovic Lauwers

**Affiliations:** 1 Department of Oral and Maxillofacial Surgery, Centre Hospitalier Universitaire (CHU) de Lille, University of Lille, Lille, FRA; 2 Department of Dento-Facial Orthopedics, Centre Hospitalier Universitaire (CHU) de Lille, University of Lille, Lille, FRA

**Keywords:** abalakov technique, mandibular distalization, oral surgery, orthodontic decompensation, skeletal anchorage

## Abstract

Orthodontic decompensation prior to orthognathic surgery often requires complex mandibular tooth movements. These movements depend on stable posterior anchorage, which is not always reliably achieved with miniscrews or miniplates. This case report describes the case of an 18-year-old patient undergoing presurgical orthodontic treatment, in whom bilateral Abalakov anchorage was performed. The technique enabled controlled distalization of the molars and retroclination of the incisors without additional skeletal hardware. Dental movements were completed in three months, with good clinical tolerance and no complications. This case illustrates an anchorage approach for achieving rapid mandibular distalization without the need for osseointegration.

## Introduction

Mandibular molar distalization is a complex orthodontic movement requiring stable anchorage to avoid unwanted side effects [1]. While miniscrews offer ease of placement, their failure rate is non-negligible. Miniplates, though more stable, involve invasive procedures and may lack patient tolerance [2-4]. The Abalakov technique, originally derived from mountaineering, involves creating a V-shaped tunnel through a solid structure to pass a looped thread for anchorage. Applied to orthodontics, this technique consists of passing a looped steel wire through the mandibular ramus, offering stable skeletal anchorage with minimal surgical complexity. This approach allows simultaneous molar distalization and incisor retroclination [5]. This case report illustrates the bilateral use of the Abalakov technique for presurgical orthodontic decompensation in preparation for orthognathic surgery. Although the Abalakov-inspired technique has been described in a few clinical cases for skeletal anchorage, its bilateral application in the mandible remains exceptional and underreported, particularly in adult orthodontic treatment where the patient seeks a rapid therapeutic approach.

## Case presentation

**Figure 1:**
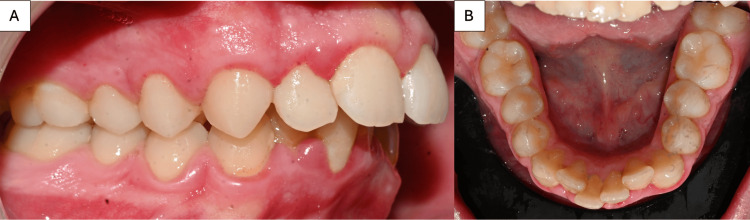
A) Preoperative intraoral view showing a dental Class II malocclusion; B) Preoperative intraoral photographs illustrating significant anterior mandibular crowding.

**Figure 2:**
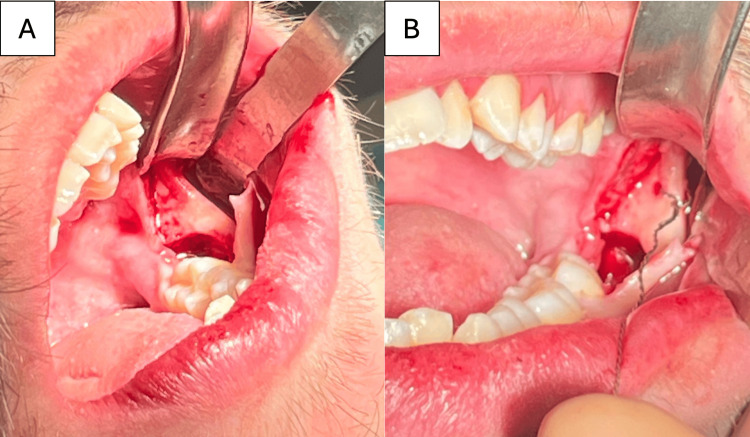
A) Intraoperative view showing bone exposure following mucoperiosteal flap elevation; B) Intraoperative photographs demonstrating drilling of the anchorage tunnel and placement of the wire (Abalakov technique).

**Figure 3:**
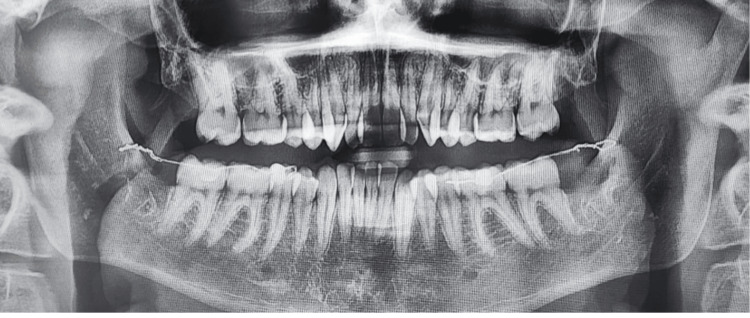
Postoperative panoramic radiograph confirming correct positioning of the bilateral Abalakov anchorage system.

**Figure 4:**
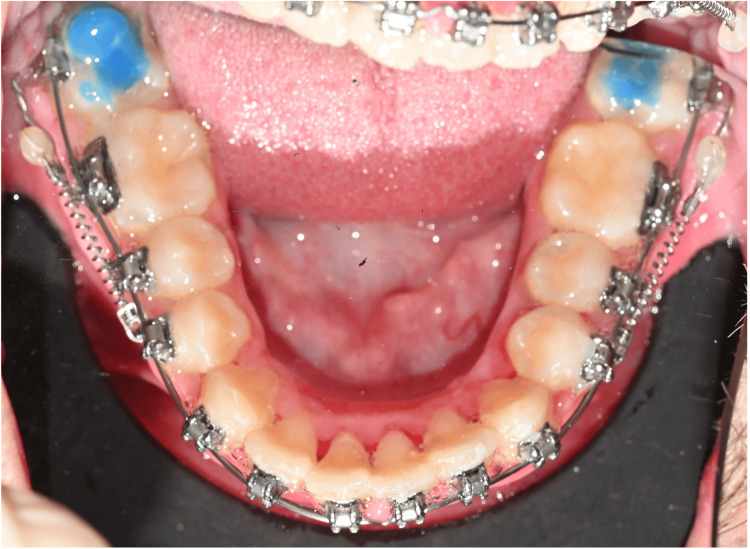
Intraoral follow-up view showing mandibular molar distalization and resolution of anterior crowding.

An 18-year-old male patient presented to the dental surgery faculty at Centre Hospitalier Universitaire (CHU) de Lille with a chief complaint of lower incisor crowding, motivated by both aesthetic and functional concerns. He had no relevant medical or dental history. Clinical examination revealed a skeletal Class II pattern with transverse maxillary deficiency and significant anterior mandibular crowding (Figure1). The orthodontic team proposed a bimaxillary ortho-surgical treatment plan aiming for alignment and leveling. To facilitate presurgical decompensation, bilateral Abalakov anchorage was used in the mandible, combined with third molar extraction. The protocol also included maxillary arch expansion and future mandibular advancement surgery. The extraction of the third molars and the placement of the bilateral Abalakov anchorage were performed in a single operative session under local anesthesia. The patient was positioned in dorsal decubitus with adequate mouth opening. A mucosal incision was made along the internal oblique ridge of the mandibular ramus. Subperiosteal dissection was performed to expose the bone. The third molars were removed first, following an alveolectomy using a round bur and corono-radicular sectioning. Two converging holes (1.6 mm) were then drilled to create a V-shaped bone tunnel. The tunnel was drilled at mid-coronal height of the mandibular crowns in order to limit any risk of extrusion or intrusion movements. Proper positioning of the entry point and controlled drill orientation (horizontal plane relative to the mandibular dental arch) are essential to preserve cortical integrity. The osteosynthesis wire was threaded through the tunnel, forming a loop that emerged intraorally (Figure 2). The wire was tightened and secured, ready for connection to the orthodontic appliance. The mucosa was repositioned and sutured. Postoperative instructions were provided. A postoperative panoramic radiograph was obtained to assess the correct positioning of the anchorage system (Figure 3). The postoperative period was uneventful, with no complications such as pain, bleeding, or inflammation. Within three months, a mandibular distalization of 2 mm was objectively achieved, sufficient to reduce anterior crowding and facilitate preparation for the mandibular advancement surgery (Figure 4).

## Discussion

The Abalakov-inspired anchorage technique was chosen in this case due to specific anatomical and clinical constraints. The patient required symmetrical bilateral distalization of the mandibular arch as part of presurgical orthodontic decompensation for Class II surgery, with a strong preference for a rapid and minimally invasive approach.

Traditional anchorage methods, such as mini-screws or miniplates, were considered less suitable due to their limitations in posterior mandibular areas, especially in non-attached gingiva, where they carry higher risks of inflammation, screw instability, and mucosal irritation. According to Grzelczyk et al. [6], mini-screws in the mandible present failure rates up to 16% to 23%, with such complications often occurring in the retromolar area. Other authors have reported failure rates for miniscrews and miniplates ranging from 10% to 20%, depending on the study, with complications including loss of anchorage, mobility, inflammation, and pain [7-9]. In contrast, Abalakov anchorage showed lower complication rates (inflammation 5.1%, infection 1.7%, anchor loss 13.6%) with treatment times averaging six months [6]. In our case, no significant postoperative complications such as pain, bleeding, or inflammation were observed. This aligns with the findings of Depeyre et al., who highlight the low morbidity of the technique compared to more invasive anchorage devices [4]. 

Moreover, the Abalakov technique allows for immediate loading and provides deep posterior anchorage, which is difficult to achieve with miniscrews or miniplates. These advantages made it particularly appropriate in this case, where stable, efficient, and well-tolerated anchorage was required without resorting to osteointegrated devices. It is also cost-effective, relying on widely available materials such as osteosynthesis wire, in contrast to more expensive and technically demanding alternatives like miniplates or miniscrews.

A key benefit of this technique lies in the possibility of immediate loading, which facilitates the early application of orthodontic forces. In our case, a 2 mm distalization of the mandibular molars was achieved within three months, accompanied by a substantial improvement in anterior crowding. Similarly, authors reported an average distal movement of 2.8 mm of the mandibular first molar crown after six months of treatment, with a corono-distal tipping of 7.5°, confirming the potential of this technique to achieve effective dental movements within a relatively short timeframe [3].

Clinically, the Abalakov technique proves particularly relevant in skeletal Class II discrepancies, especially when integrated into an orthognathic surgery protocol. It enables efficient presurgical orthodontic decompensation while avoiding the need for additional skeletal hardware.

This case is original in that it describes a bilateral application of the Abalakov technique in a young adult patient within the specific context of pre-surgical orthodontic decompensation for Class II surgery. The technique was chosen to meet the need for rapid mandibular distalization using a minimally invasive intraoral approach, without resorting to osteointegrated devices. To our knowledge, no prior reports have described this specific combination of clinical indications and technique.

However, some limitations must be acknowledged. Anatomic variability of the mandibular ramus can affect the feasibility and symmetry of the technique. Moreover, successful outcomes depend heavily on surgical precision. Incorrect angulation of the bone tunnels or improper wire tensioning can compromise anchorage stability. As Bernard-Granger et al. suggest, specific training in this technique is advisable to ensure consistent and reliable outcomes [5].

## Conclusions

The Abalakov technique provides a minimally invasive and accessible skeletal anchorage option for presurgical orthodontic treatment. It allows for efficient molar distalization and incisor alignment with minimal complications. Although miniscrews and miniplates are widely used for skeletal anchorage, both techniques have been associated with various postoperative complications such as soft tissue irritation, inflammation, screw loosening or failure, and patient discomfort, particularly in the posterior mandibular region. In contrast, the Abalakov-inspired approach allowed for stable and effective anchorage with minimal invasiveness and excellent patient tolerance. In our case, 2 mm of molar movement was achieved in just three months. Its simplicity, cost-efficiency, and use under local anesthesia make it a valuable alternative to conventional anchorage systems. Nevertheless, anatomical variability and surgical precision remain critical. Further research is needed to validate its long-term stability and broader clinical application.
